# 5-Bromo-*N*-methyl­pyrimidin-2-amine

**DOI:** 10.1107/S1600536811051531

**Published:** 2011-12-14

**Authors:** Qi Yang, Ning Xu, Kai Zhu, Xiaoping Lv, Ping-fang Han

**Affiliations:** a College of Life Science and Pharmaceutical Engineering, Nanjing University of Technology, Xinmofan Road No. 5 Nanjing, Nanjing 210009, People’s Republic of China; b College of Environment, Nanjing University of Technolgy, Xinmofan Road No. 5 Nanjing, Nanjing 210009, People’s Republic of China

## Abstract

In the title mol­ecule, C_5_H_6_BrN_3_, the pyrimidine ring is essentially planar, with an r.m.s. deviation of 0.007 Å. The Br and N atoms substituted to the pyrimidine ring are coplanar with the ring [displacements = 0.032 (1) and 0.009 (5) Å, respectively], while the methyl C atom lies 0.100 (15) Å from this plane with a dihedral angle between the pyrimidine ring and the methyl­amine group of 4.5 (3)°. In the crystal, C—H⋯N, C—H⋯Br and N—H⋯N hydrogen bonds link the mol­ecules into a two-dimensional network in the (011) plane.

## Related literature

Derivatives of pyrimidine are important chemical materials, see: Yu *et al.* (2007 ▶). For a related structure, see: Aakeroey *et al.* (2005 ▶).
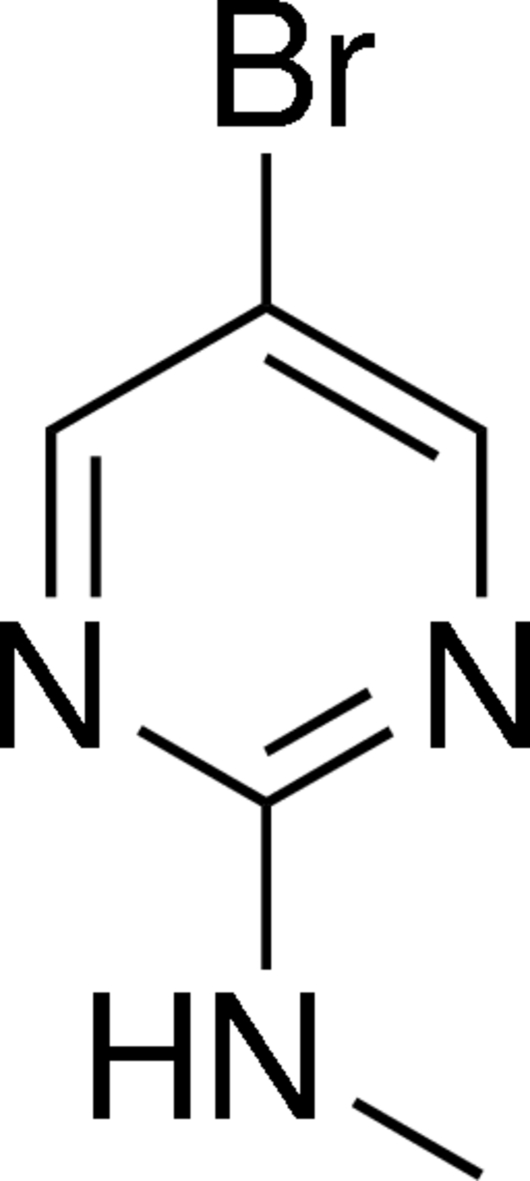


## Experimental

### 

#### Crystal data

C_5_H_6_BrN_3_
*M* *_r_* = 188.04
Triclinic, 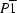

*a* = 3.9900 (8) Å
*b* = 9.862 (2) Å
*c* = 10.006 (2) Å
α = 61.57 (3)°
β = 83.84 (3)°
γ = 87.45 (3)°
*V* = 344.24 (16) Å^3^
*Z* = 2
Mo *K*α radiation
μ = 5.88 mm^−1^
*T* = 293 K
0.10 × 0.05 × 0.05 mm

#### Data collection

Enraf–Nonius CAD-4 diffractometer
Absorption correction: ψ scan (North *et al.*, 1968 ▶) *T*_min_ = 0.591, *T*_max_ = 0.758
1454 measured reflections
1260 independent reflections
714 reflections with *I* > 2σ(*I*)
*R*_int_ = 0.089
3 standard reflections every 200 reflections  intensity decay: 1%

#### Refinement

*R*[*F* ^2^ > 2σ(*F* ^2^)] = 0.056
*wR*(*F* ^2^) = 0.100
*S* = 1.00
1260 reflections
82 parameters
H-atom parameters constrained
Δρ_max_ = 0.40 e Å^−3^
Δρ_min_ = −0.39 e Å^−3^

### 

Data collection: *CAD-4 Software* (Enraf–Nonius, 1989 ▶); cell refinement: *CAD-4 Software*; data reduction: *XCAD4* (Harms & Wocadlo, 1995 ▶); program(s) used to solve structure: *SHELXS97* (Sheldrick, 2008 ▶); program(s) used to refine structure: *SHELXL97* (Sheldrick, 2008 ▶); molecular graphics: *PLATON* (Spek, 2009 ▶); software used to prepare material for publication: *SHELXL97*.

## Supplementary Material

Crystal structure: contains datablock(s) global, I. DOI: 10.1107/S1600536811051531/pv2488sup1.cif

Structure factors: contains datablock(s) I. DOI: 10.1107/S1600536811051531/pv2488Isup2.hkl

Supplementary material file. DOI: 10.1107/S1600536811051531/pv2488Isup3.cml

Additional supplementary materials:  crystallographic information; 3D view; checkCIF report

## Figures and Tables

**Table 1:** Hydrogen-bond geometry (Å, °)

*D*—H⋯*A*	*D*—H	H⋯*A*	*D*⋯*A*	*D*—H⋯*A*
N1—H1*A*⋯N2^i^	0.86	2.19	3.035 (7)	169
C1—H1*B*⋯Br^ii^	0.96	2.85	3.751 (8)	157
C5—H5*A*⋯N3^iii^	0.93	2.59	3.357 (7)	140

Symmetry codes: (i) 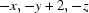; (ii) 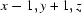; (iii) 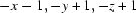.

**Fig. 1.**
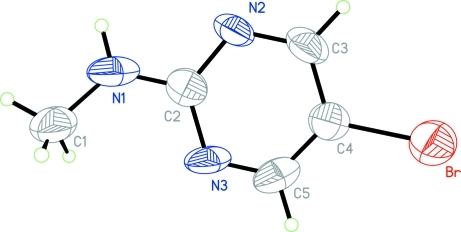
The molecular structure of the title compound showing atom-numbering scheme and displacement ellipsoids plotted at 30% probability level.

**Fig. 2.**
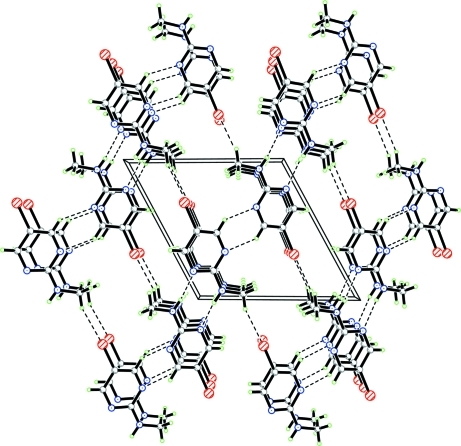
A packing diagram of the title compound. The intermolecular hydrogen bonding interactions are shown as dashed lines.

## supplementary crystallographic information

### Comment

Some derivatives of pyrimidin are important chemical materials (Yu *et al.*, 2007). Here in this article, the preparation and crystal structure of the title compound is presented. The pyrimidin ring is essentially planar with rms deviation 0.0071. The atoms Br and N1 are coplanar with the pyrimidin ring while C1 lies 0.100 (15) Å from this plane with a dihedral angle between the pyrimidin ring and the methylamine group 4.5 (3)°. In the crystal structure, intermolecular C—H···N, C—H···Br and N—H···N hydrogen bonding interactions link the molecules into a two dimensional cluster in (0 1 1) plane (Tab. 1 and Fig. 2).

### Experimental

5-Bromo-hexahydro-pyrimidine (2.06 g, 0.01 mol) and 1,3-propanediamine (1.48 g, 0.02 mol) were refluxed in 10 ml benzene for 18 h. After completion of the reaction (TLC control), the product was washed with cold toluene (2*15 ml), at room temperature, dried over sodium sulfate and yielded 2.43 g (69%) of the title compound which was further purified by crystallization from methanol. Crystals of the title compound suitable for X-ray crystallographic studies were obstained by slow evaporation of a methanol solution.

### Refinement

H atoms were positioned geometrically, with N—H = 0.86 Å and C—H = 0.93 and 0.96 Å for aryl and methyl H atoms, respectively, and constrained to ride on their parent atoms, with *U*_iso_(H) = 1.2*U*_eq_(N/C-aryl) or 1.5*U*_eq_(C-methyl).

### Crystal data

**Table:**

C_5_H_6_BrN_3_	*Z* = 2
*M**_r_* = 188.04	*F*(000) = 184
Triclinic, *P*1	*D*_x_ = 1.814 Mg m^−^^3^
Hall symbol: -P 1	Mo *K*α radiation, λ = 0.71073 Å
*a* = 3.9900 (8) Å	Cell parameters from 25 reflections
*b* = 9.862 (2) Å	θ = 9–14°
*c* = 10.006 (2) Å	µ = 5.88 mm^−^^1^
α = 61.57 (3)°	*T* = 293 K
β = 83.84 (3)°	Block, colorless
γ = 87.45 (3)°	0.10 × 0.05 × 0.05 mm
*V* = 344.24 (16) Å^3^	

### Data collection

**Table:**

Enraf–Nonius CAD-4 diffractometer	714 reflections with *I* > 2σ(*I*)
Radiation source: fine-focus sealed tube	*R*_int_ = 0.089
graphite	θ_max_ = 25.4°, θ_min_ = 2.3°
ω/2θ scans	*h* = 0→4
Absorption correction: ψ scan (North *et al.*, 1968)	*k* = −11→11
*T*_min_ = 0.591, *T*_max_ = 0.758	*l* = −11→11
1454 measured reflections	3 standard reflections every 200 reflections
1260 independent reflections	intensity decay: 1%

### Refinement

**Table:**

Refinement on *F*^2^	Primary atom site location: structure-invariant direct methods
Least-squares matrix: full	Secondary atom site location: difference Fourier map
*R*[*F*^2^ > 2σ(*F*^2^)] = 0.056	Hydrogen site location: inferred from neighbouring sites
*wR*(*F*^2^) = 0.100	H-atom parameters constrained
*S* = 1.00	*w* = 1/[σ^2^(*F*_o_^2^) + (0.0385*P*)^2^] where *P* = (*F*_o_^2^ + 2*F*_c_^2^)/3
1260 reflections	(Δ/σ)_max_ < 0.001
82 parameters	Δρ_max_ = 0.40 e Å^−^^3^
0 restraints	Δρ_min_ = −0.39 e Å^−^^3^

### Special details

**Table:**

Geometry. All e.s.d.'s (except the e.s.d. in the dihedral angle between two l.s. planes) are estimated using the full covariance matrix. The cell e.s.d.'s are taken into account individually in the estimation of e.s.d.'s in distances, angles and torsion angles; correlations between e.s.d.'s in cell parameters are only used when they are defined by crystal symmetry. An approximate (isotropic) treatment of cell e.s.d.'s is used for estimating e.s.d.'s involving l.s. planes.
Refinement. Refinement of *F*^2^ against ALL reflections. The weighted *R*-factor *wR* and goodness of fit *S* are based on *F*^2^, conventional *R*-factors *R* are based on *F*, with *F* set to zero for negative *F*^2^. The threshold expression of *F*^2^ > σ(*F*^2^) is used only for calculating *R*-factors(gt) *etc*. and is not relevant to the choice of reflections for refinement. *R*-factors based on *F*^2^ are statistically about twice as large as those based on *F*, and *R*- factors based on ALL data will be even larger.

### Fractional atomic coordinates and isotropic or equivalent isotropic displacement parameters (Å^2^)

**Table:**

	*x*	*y*	*z*	*U*_iso_*/*U*_eq_	
Br	0.15554 (17)	0.32133 (9)	0.25400 (8)	0.0790 (4)	
N1	−0.2779 (12)	0.9216 (6)	0.1902 (5)	0.0673 (15)	
H1A	−0.2201	1.0018	0.1048	0.081*	
C1	−0.4717 (15)	0.9457 (7)	0.3050 (7)	0.080 (2)	
H1B	−0.5157	1.0538	0.2663	0.120*	
H1C	−0.3489	0.9094	0.3930	0.120*	
H1D	−0.6814	0.8903	0.3330	0.120*	
N2	0.0176 (11)	0.7834 (5)	0.0874 (5)	0.0545 (13)	
C2	−0.1772 (14)	0.7836 (7)	0.2042 (7)	0.0494 (15)	
N3	−0.2888 (11)	0.6545 (6)	0.3321 (5)	0.0549 (13)	
C3	0.1196 (13)	0.6469 (7)	0.1010 (7)	0.0592 (16)	
H3A	0.2605	0.6407	0.0239	0.071*	
C4	0.0086 (14)	0.5125 (7)	0.2352 (7)	0.0535 (16)	
C5	−0.1934 (14)	0.5251 (7)	0.3455 (7)	0.0574 (17)	
H5A	−0.2648	0.4360	0.4340	0.069*	

### Atomic displacement parameters (Å^2^)

**Table:**

	*U*^11^	*U*^22^	*U*^33^	*U*^12^	*U*^13^	*U*^23^
Br	0.0629 (5)	0.0737 (5)	0.0811 (6)	−0.0023 (3)	0.0026 (3)	−0.0229 (4)
N1	0.059 (3)	0.064 (4)	0.049 (3)	−0.015 (3)	0.015 (3)	−0.006 (3)
C1	0.082 (5)	0.050 (4)	0.077 (5)	−0.001 (3)	0.037 (4)	−0.015 (4)
N2	0.048 (3)	0.061 (3)	0.042 (3)	0.003 (2)	0.015 (2)	−0.019 (3)
C2	0.049 (4)	0.060 (4)	0.042 (4)	0.000 (3)	−0.009 (3)	−0.025 (3)
N3	0.048 (3)	0.049 (3)	0.041 (3)	−0.013 (2)	0.013 (2)	−0.002 (3)
C3	0.039 (3)	0.074 (4)	0.058 (4)	−0.001 (3)	0.013 (3)	−0.028 (4)
C4	0.046 (4)	0.061 (4)	0.047 (4)	0.004 (3)	−0.006 (3)	−0.020 (3)
C5	0.054 (4)	0.044 (4)	0.053 (4)	−0.010 (3)	−0.006 (3)	−0.005 (3)

### Geometric parameters (Å, °)

**Table:**

Br—C4	1.876 (6)	N2—C3	1.336 (7)
N1—C2	1.347 (7)	C2—N3	1.354 (7)
N1—C1	1.424 (7)	N3—C5	1.264 (7)
N1—H1A	0.8600	C3—C4	1.409 (8)
C1—H1B	0.9600	C3—H3A	0.9300
C1—H1C	0.9600	C4—C5	1.347 (8)
C1—H1D	0.9600	C5—H5A	0.9300
N2—C2	1.333 (7)		
			
C2—N1—C1	125.5 (5)	N1—C2—N3	118.5 (5)
C2—N1—H1A	117.2	C5—N3—C2	118.5 (5)
C1—N1—H1A	117.2	N2—C3—C4	118.4 (6)
N1—C1—H1B	109.5	N2—C3—H3A	120.8
N1—C1—H1C	109.5	C4—C3—H3A	120.8
H1B—C1—H1C	109.5	C5—C4—C3	119.4 (6)
N1—C1—H1D	109.5	C5—C4—Br	122.4 (5)
H1B—C1—H1D	109.5	C3—C4—Br	118.1 (5)
H1C—C1—H1D	109.5	N3—C5—C4	121.9 (5)
C2—N2—C3	117.5 (5)	N3—C5—H5A	119.1
N2—C2—N1	117.3 (5)	C4—C5—H5A	119.1
N2—C2—N3	124.2 (6)		
			
C3—N2—C2—N1	179.6 (5)	C2—N2—C3—C4	1.7 (8)
C3—N2—C2—N3	−2.9 (8)	N2—C3—C4—C5	−0.5 (9)
C1—N1—C2—N2	−176.7 (6)	N2—C3—C4—Br	−179.6 (4)
C1—N1—C2—N3	5.7 (8)	C2—N3—C5—C4	−1.4 (9)
N2—C2—N3—C5	2.8 (8)	C3—C4—C5—N3	0.4 (9)
N1—C2—N3—C5	−179.7 (5)	Br—C4—C5—N3	179.4 (4)

### Hydrogen-bond geometry (Å, °)

**Table:**

*D*—H···*A*	*D*—H	H···*A*	*D*···*A*	*D*—H···*A*
N1—H1A···N2^i^	0.86	2.19	3.035 (7)	169.
C1—H1B···Br^ii^	0.96	2.85	3.751 (8)	157.
C5—H5A···N3^iii^	0.93	2.59	3.357 (7)	140.

Symmetry codes: (i) −*x*, −*y*+2, −*z*; (ii) *x*−1, *y*+1, *z*; (iii) −*x*−1, −*y*+1, −*z*+1.
